# Splicing to keep splicing: A feedback system for cellular homeostasis and state transition

**DOI:** 10.1002/ctm2.70369

**Published:** 2025-06-19

**Authors:** Zhonghao Guo, Xurui Zhang, Yachen Li, Yule Chen, Yungang Xu

**Affiliations:** ^1^ Department of Urology the First Affiliated Hospital of Xi'an Jiaotong University Xi'an China; ^2^ Department of Cell Biology and Genetics School of Basic Medical Sciences Xi'an Jiaotong University Health Science Center Xi'an China; ^3^ Department of Epidemiology Biostatistics and Occupational Health School of Population and Global Health McGill University Montreal Canada

**Keywords:** alternative splicing, autoregulation, feedback, non‐coding RNAs

## Abstract

**Background:**

Alternative splicing (AS) plays a crucial role in regulating gene expression and governing proteomic diversity by generating multiple protein isoforms from a single gene. Increasing evidence has highlighted the regulation for pre‐mRNA splicing of the splicing factors (SFs). This review aims to examine featured mechanisms and examples of SF regulation by AS, focusing on paradigmatic feedback loops and their biological implications.

**Main Body of the Abstract:**

We specifically focus on the autoregulation and inter‐regulation of SFs through AS machinery. These interactions give rise to a feedback system, where the negative feedback loops aid in maintaining cellular homeostasis, and the positive feedback loops play roles in triggering cellular state transitions. We examine the growing evidence highlighting the specific mechanisms employed by SFs to autoregulate their own splicing, including AS‐coupled nonsense‐mediated mRNA decay (AS‐NMD), nuclear retention, and alternative 3'UTR regulation. We showcase the influence of AS feedback in amyotrophic lateral sclerosis (ALS), frontotemporal dementia (FTD), and cancer. Furthermore, we discuss how master splicing factors can dominantly orchestrate splicing cascades, leading to widespread impacts in cellular processes. We also discuss how non‐coding RNAs, particularly circular RNAs and microRNAs, engage in the splicing regulatory networks. Lastly, we showcase how negative and positive feedback loops can collaboratively achieve remarkable biological functions during the cell fate decision.

**Short Conclusion:**

This review highlights the regulation of SFs by AS, providing enriched information for future investigations that aim at deciphering the intricate interplay within splicing regulatory networks.

**Key Points:**

- Negative feedback of alternative splicing maintains cellular homeostasis.
- Positive feedback of alternative splicing triggers cellular state transitions.
- Alternative splicing forms integrated feedback networks with circRNAs and microRNAs to reciprocally regulate their expression and function.
- The coordinated interplay of distinct splicing feedback mechanisms orchestrates precise cell fate transitions.
- Future directions and therapeutic possibilities that could transform alternative splicing research into treatments.

## 1 INTRODUCTION

Splicing is a key process in the maturation of eukaryotic mRNA, which involves the removal of introns from the pre‐mRNA and concatenation of exons.^1^ The eukaryotic genes often allow different combinations of exons to be included or excluded, a phenomenon known as alternative splicing (AS), enabling a single gene to produce multiple mRNA transcripts and, consequently, diverse protein isoforms.^2^ AS plays a crucial role in various biological processes, including (i) development and physiology,^3^ (ii) establishment of tissue identity,^4^ and (iii) disease onset when disrupted.^5^ The intricate regulation of AS is accurately orchestrated by trans‐acting splicing factors (SFs), which bind to sequence motifs (*cis*‐acting elements) that either enhance or repress splicing.^6^ SFs can be basically classified into three common types: serine/arginine‐rich (SR) proteins, heterogeneous nuclear ribonucleoproteins (hnRNPs), and tissue‐specific SFs.^7^ Mutations of SFs or dysregulation of their gene expression can result in several human cancers and other genetic disorders.

It was only after the advent of next‐generation sequencing and transcript assembly tools that biologists realised that AS was a common occurrence rather than an anomaly in multicellular organisms, taking place in about 95% of human genes.^3^ Since then, research on AS has advanced from observing individual splicing events and their effects on protein expression to characterising comprehensive AS networks and their coordination.^4^ In the last decade, an increasing number of studies have been attempting to unravel the following questions: how does a specific SF engage in its own mRNA splicing to regulate its expression and isoform ratio? How do SFs mutually control the splicing of one another to amplify and convey the regulatory influence of AS? What are the repercussions of this feedback loop or cascade on the maintenance of physiological processes or the progression of diseases when dysregulated? While numerous cases of splicing autoregulation and inter‐regulation have been reported, a focused synthesis of representative feedback systems and their roles in cellular function and disease remains valuable.

Therefore, in this focused review, we delve deeply into the complex regulatory framework of AS, concentrating specifically on how SFs regulate themselves and each other through AS mechanisms. These interactions create feedback loops, both negative ones that help maintain cellular balance and positive ones that drive cellular transitions. We explore the mounting evidence detailing how SFs control their own splicing, utilising processes like AS‐coupled nonsense‐mediated mRNA decay (AS‐NMD), nuclear retention, and alternative 3'UTR regulation. Additionally, we examine how key SFs can dictate entire splicing cascades, profoundly impacting various cellular functions. We also explore the involvement of non‐coding RNAs (ncRNAs), particularly circular RNAs (circRNA) and microRNAs, in these splicing networks. Finally, we illustrate how negative and positive feedback loops work together to accomplish crucial biological functions during cellular decision‐making. This review serves as a valuable resource for future studies seeking to fully understand the intricate dynamics within splicing regulatory networks.

## 2 NEGATIVE FEEDBACK MAINTAINS HOMEOSTASIS

**FIGURE 1:**
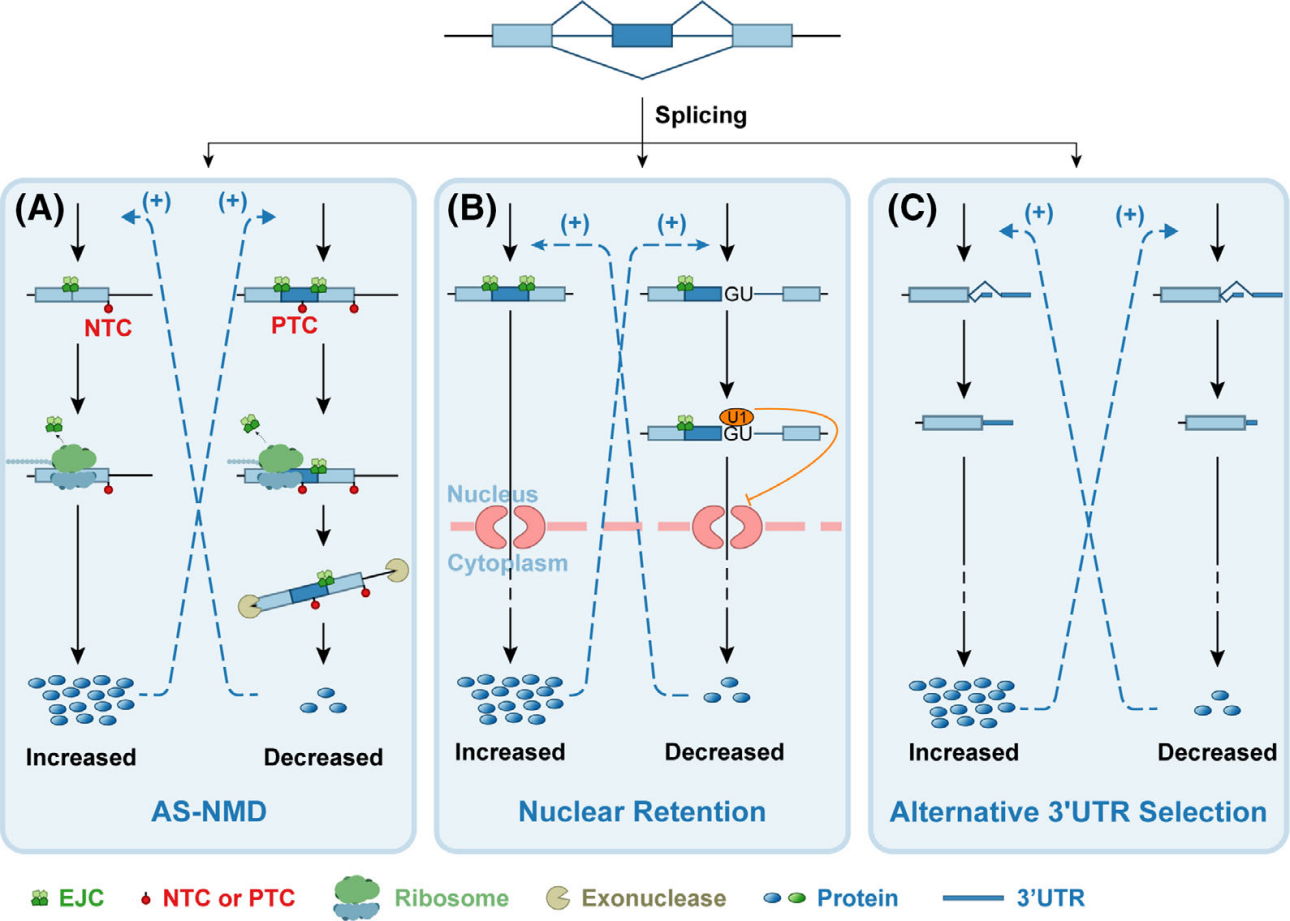
Negative feedback of alternative splicing maintains homeostasis. (A) Alternative splicing coupled with nonsense‐mediated mRNA decay (AS‐NMD) can occur when a premature termination codon (PTC) is located more than 50–54 nucleotides upstream of at least one exon‐exon junction (EJC). This would disrupt the function of the normal termination codon (NTC). This results in mRNA degradation and the production of truncated proteins. The retention of a PTC‐containing exon is one of the mechanisms that could induce AS‐NMD. AS‐NMD, Alternative splicing coupled with nonsense‐mediated mRNA decay; PTC, premature termination codon; EJC, exon junction complex; NTC, normal termination codon. (B) Nuclear retention can occur when introns remain in mRNA, leading to the presence of intact 5′ splice site (5′SS) motifs. These motifs can recruit U1 snRNP, thereby preventing the export of mRNA from the nucleus to the cytoplasm. 5′SS, 5′ splice site. (C) Protein isoforms are regulated through various mechanisms such as AS‐NMD, nuclear retention, alternative 3'UTR, and non‐coding RNA interference. Isoforms translated from mRNAs with different exons and 3'UTRs were illustrated in the figure as an example. Typically, in negative feedback, one isoform promotes the splicing of its counterpart while inhibiting its own splicing. Assuming a constant abundance of the primary transcript, this can help achieve a balance between the two isoforms.

Negative feedback is a pivotal biological regulatory mechanism where the end product of a process reduces the stimulus that triggers that same process, thereby maintaining homeostasis. Specifically in AS, negative feedback regulation of SFs can occur through distinct mechanisms: at the RNA level, SFs may (1) go through the nonsense‐mediated mRNA decay (NMD)^8^; (2) be blocked against translation due to nuclear retention^9^; (3) bind to the 3′‐untranslated regions (3′UTRs) of their own transcripts and destabilise them.^10^ At the protein level, SFs can (4) be enzymatically modified by downstream products.^11^ The three most commonly observed mechanisms are AS‐NMD, nuclear retention, and alternative 3'UTR regulation, as depicted in Figure 1. It is worth noting that multiple mechanisms can combine and participate in the feedback regulation of one single gene, such as the scenario reported by the studies on TDP‐43.^12^, ^13^

### 2.1 Alternative splicing coupled nonsense‐mediated mRNA decay

Nonsense‐mediated mRNA decay (NMD) is a surveillance mechanism in the cytoplasm that recognises and degrades mRNAs with premature termination codons (PTCs) to prevent the production of truncated proteins that could lead to abnormalities.^14^ Specifically, the NMD pathway is guided by the position of the termination codon relative to the exon junction complex (EJC).^15^ The EJC is a multiprotein complex that is deposited by spliceosomes 24 nucleotides upstream of the spliced junctions of mRNA.^16^ Normally, when a normal termination codon (NTC) is located on the last exon, full‐length functional proteins can be produced. However, if a PTC is situated more than 50–54 nucleotides upstream of at least one exon–exon junction, the ribosome is unable to displace the distal EJC(s).^15^ Consequently, the retained EJC(s) will initiate the NMD pathway by recruiting core NMD factors, including UPF1, UPF2, UPF3B, and the kinase SMG1, which collectively mediate mRNA surveillance and degradation.^15^ Although PTCs can arise from gene mutations, AS can also introduce PTCs into mRNAs, known as alternative splicing coupled nonsense‐mediated mRNA decay (AS‐NMD) (see Figure 1A). The AS events that could induce AS‐NMD include: frameshift due to exon skipping or intron retention; splicing in the 3'UTR, at a position located ≥ 55 nucleotides downstream of the normal stop codon, creating a premature context; retention of a PTC‐containing exon (known as poison cassette exon, PCE); and retention of the intron with an in‐frame PTC.^17^ Studies have found that up to one‐third of human AS events contain PTCs, and a total of 21,384 transcripts are annotated as NMD candidates.^18^ This widespread presence allows NMD to maintain 10%–20% normal protein levels under physiological conditions.^15^ The splicing process that targeted towards NMD can be induced by overexpression of truncated proteins, protein isoforms, or normal proteins, operating as a feedback mechanism.^12^, ^17^ One classic scenario is that SFs can regulate their own levels. For example, most SR mRNAs have shown the possibility of PTC formation, which could further trigger the NMD of themselves.^17^, ^19^ SRSF3 regulates its own expression by promoting the inclusion of exon 4, which contains a PTC and results in the production of truncated SRSF3.^8^ PTCs involved in AS‐NMD, especially all PTCs within the SR protein family, are highly conserved across species.^20^

Prevention of AS‐NMD can be accomplished by blocking the binding site of NMD factors on mRNAs. One example is the binding of PTBP1 (polypyrimidine tract‐binding protein 1) with an RNA stability element (RSE) in transcripts, which obstructs the binding of UPF1, an NMD protein, to the 3'UTRs of these transcripts.^21^, ^22^ This mechanism was originally discovered in respiratory syncytial virus (RSV) as a means of evading degradation by the host cell's NMD machinery. However, many human transcripts that have long 3’UTRs also use this strategy to maintain their stability.^21^ The increased concentration of high‐affinity PTBP1‐ or hnRNP L‐binding sites in the 3′UTR can also attract these SFs and hinder UPF1 translocation, thereby preventing NMD.^23^ A study on the autoregulation of SRSF7 suggests that split‐ORFs, bicistronic transcripts encoding truncated proteins, can block AS‐NMD by promoting intron retention and preventing poison cassette exon (containing a PTC) inclusion. Specifically, while transient overexpression of SRSF7 can trigger AS‐NMD to reduce SRSF7 level, sustained overexpression of SRSF7 can result in SRSF7 transcripts with unspliced introns 3 and 5. These intron‐retained transcripts act as architectural RNAs (arcRNAs), which contain repeated SRSF7 binding motifs. Through massive SRSF7 binding and oligomerisation via its RS domain, these arcRNAs assemble phase‐separated nuclear condensates that sequester SRSF7 protein, reducing its functional availability in the nucleus and limiting the cytoplasmic pool of translatable SRSF7 mRNA. This mechanism turns out to be a stronger and faster way to regulate SRSF7 level than AS‐NMD.^24^ Another interesting situation is monoallelic mutation, where one allele of a gene is nonfunctional due to a mutation, while the other allele remains functional. Theoretically, in the absence of feedback modulation, the expression of the wild‐type protein in a heterozygous (±) genomic context would be expected to be 50% of that in a homozygous wild‐type (+/+) context. However, a recent study examining various heterozygous mutations in the *PHF5A* gene found that levels of the wild‐type PHF5A protein ranged from 60% to 100%. Additionally, the transcripts of the mutated PHF5A, which contain a PTC, were found to be stable and not subject to NMD. PHF5A is a crucial component of the SF3B splicing complex, and the formation of the SF3B complex remained unaffected in subjects with heterozygous *PHF5A* mutations. These findings collectively suggest the existence of a feedback system in heterozygous *PHF5A* gene that regulates NMD and ensures the maintenance of PHF5A expression.^25^ Further and broader validation is still required to understand how heterozygous situations (e.g., monoallelic mutations, chromosomal deficiencies, genomic imprinting, etc.) affect the feedback regulation of AS in a quantitative manner.

Besides serving as a passive feedback mechanism, a recent study on actin‐related protein 5 (ARP5) in mouse and human muscle cells found that AS‐NMD can also serve as an active behaviour to adjust cell differentiation, by regulating a novel ARP5 isoform that is targeted by NMD.^26^ RBM10 regulates its own mRNA as well as that of RBM5 via AS‐NMD, indicating its role beyond autoregulation.^27^ Mutations of certain SFs can alter the activity and specificity of NMD. A study found that the demethylation of 3′UTR CpGs reduces the efficiency of TDP‐43 autoregulation by attenuating NMD‐targeted AS events and increasing the canonical mRNA levels, as observed in ALS.^28^ This is consistent with previous findings that epigenetic modifications may also be involved in the process of AS‐NMD.^29^

### 2.2 Nuclear retention

The export of mRNAs from the nucleus to the cytoplasm is a crucial step in eukaryotic gene expression.^30^ Most models assume that an mRNA must have specialised signals to be exported from the nucleus.^31^ AS, in certain scenarios, promotes nuclear export by facilitating the recruitment of export factors to mature RNA with the help of spliceosomes.^32^ It is probable that mRNAs with retained introns are targeted for nuclear retention because they contain intact 5′ splice site (5′SS) motifs, either in the coding region or the 3′ untranslated region (3′UTR).^32^, ^33^ These motifs can recruit U1 snRNP, which then prevents nuclear export (see Figure 1B).^32^, ^33^ It also makes sense that the EJC is a main factor for enhancing the nuclear export of spliced mRNAs.^34^ One case is that the nuclear pool of Clk1/4 RNAs can help regulate the phosphorylation of two SFs, SRSF4 and SRSF10. *Clk1/4* RNAs are normally localised in the nucleus in an intron‐retaining form. Stress can cause the dephosphorylation of SR proteins, which leads to stress‐responsive splicing of these intron‐retaining Clk1/4 RNAs and enabling translation. Subsequently, abundant Clk1/4 proteins will rephosphorylate the SR proteins back to normal.^11^ Thus, Clk1/4 functions as a guardian, preserving the phosphorylation of SR proteins during times of stress. This also reveals a novel mechanism by which SFs can autoregulate themselves via enzymatic modification. The retention of SFs’ own mRNAs within the nucleus can also influence their functionality. Since SFs exert their effects exclusively within the nucleus, by operating on the availability of nuclear export, they can adjust their abundance in the nucleus and thus adjust their capacity of splicing. For example, as an autoregulating SF, MBNL can determine whether to skip its exon 5 or exon 7, which are essential for maintaining the integrity of the nuclear localisation signal (NLS), to regulate its presence in the nucleus.^9^, ^35^ Additionally, the presence of the 5′SS motifs in the 3′UTR promotes nuclear retention.^32^ The essential U11/U12‐65K protein component of the minor spliceosome can splice its own mRNA to produce a long 3′UTR sequence containing a tandem repeat of 5′SS sites termed USSE, which promotes retention of this isoform in the nucleus.^30^ Nuclear speckles are dynamic subnuclear domains highly enriched in pre‐mRNA SFs and other proteins involved in mRNA processing and export.^36^ Some studies have shown that certain unspliced transcripts are retained in nuclear speckles, where they are protected from degradation. Therefore, it is proposed that storing transcripts in nuclear speckles operates as a temporal control mechanism to allow mRNA export and translation at certain times, which is especially useful during cell differentiation.^37^ A study on Arabidopsis potentially suggests that transcripts retention in speckles can contribute to splicing feedback, but solid evidence on this topic is still lacking.^38^

Research indicates that in neurodegenerative diseases, nuclear export is often disrupted by aggregating cytoplasmic proteins.^39^ Amyotrophic lateral sclerosis (ALS) and frontotemporal dementia (FTD) are two diseases that form a broad neurodegenerative continuum.^40^ Pathological accumulation of TDP‐43 and FUS are hallmarks in ALS and FTD.^40^, ^41^, ^42^ Normally, TDP‐43 and FUS share similar autoregulatory mechanisms to keep their abundance steady, including AS‐NMD^13^, ^41^, ^43^, ^44^ and transcript retention in the nucleus.^12^, ^45^, ^46^, ^47^, ^48^ Persistent damage to any of these mechanisms can lead to an elevation in canonical isoforms, thereby contributing to the development of ALS/FTD. For example, in ALS, the lack of nuclear TDP‐43 triggers abnormal autoregulation, leading to an increased level of TARDBP mRNA (encoding TDP‐43).^49^ A recent study found that in ALS and FTD, acetylation and RNA‐binding ablation both enhance the propensity for TDP‐43 aggregation.^46^ This leads to the formation of pathological TDP‐43, which will sequester normal TDP‐43 to make it immobile and insoluble. The absence of functional TDP‐43 leads to increased TARDBP expression due to negative feedback, but merely resulting in more pathological TDP‐43.^46^ FUS can negatively regulate its own transcript in two ways: either by skipping exon 7 to undergo AS‐NMD or by retaining both introns 6 and 7 to detain the transcript in the nucleus and prevent translation in the cytoplasm.^41^, ^48^ In cells with FUS NLS mutations, mutant FUS cannot be effectively transported into the cell nucleus, thereby preventing it from reducing FUS translation through the latter mechanism.^48^

### 2.3 Regulations on 3′‐untranslated regions

Research has shown that 3′UTRs control mRNA stability, translation efficiency, and localisation.^50^ Therefore, AS of the 3'UTR is unlikely to alter the protein product itself, but rather its abundance in a quantitative manner.^10^ The deep sequencing of 3′‐end has revealed that over half of human and mouse genes generate alternative 3′UTR mRNA isoforms.^8^ These isoforms are produced by two mechanisms collaboratively: alternative polyadenylation, which involves cleavage at different sites within the 3'UTR, and AS, which affects the introns present in the 3'UTR.^51^ It was traditionally known that alternative 3′UTRs produced from a gene are interconnected, meaning that increasing the expression of one isoform will decrease the expression of the other isoform, assuming gene expression levels remain constant^51^ (see Figure 1C). Changes in 3′UTR length alone due to splicing could alter several regulatory elements. Specifically, 3′UTR shortening is often suggested to enhance mRNA stability and translational efficiency by reducing the number of regulatory elements—such as miRNA‐ and RBP‐binding sites—that can mediate mRNA degradation or translational repression. However, the impact of 3′UTR length is context‐dependent, and longer 3′UTRs can, in some cases, also promote mRNA stability or translation depending on the presence of specific regulatory elements.^52^ Moreover, it can also lead to NMD, as long as a termination codon exists ≥ 55 nucleotides upstream of a splice junction.^53^ Given the many cases where alternative 3′UTR transcripts can bypass NMD and undergo translation, it is plausible that certain 3′UTR‐related factors can inhibit NMD.^8^ For example, these factors may act by obstructing the recruitment of UPF1, an NMD factor that accumulates in the 3′UTR.^21^, ^23^

Several studies have demonstrated AS of 3′UTR as a mechanism for autoregulation. The PAR‐2 domain size is a major factor associated with cell polarity in *C. elegans*, and it is regulated by expression of the PAR‐5 level.^54^ PAR‐5 level and its isoform ratio are tightly controlled by AS of *par‐5* 3′UTR. Specifically, *par‐5::utr.1* and *par‐5::utr.2* are shown to have longer 3′UTR and higher translational activity. In *par‐2* mutants, impaired polarity can feed back to relatively increase *par‐5::utr.1* and *par‐5::utr.2*, and this selection of *par‐5* isoform also contributes to the robustness of polarity.^10^, ^55^ The previous section discussed ALS/FTD resulting from defects in nuclear retention feedback. Notably, this process can also be impaired by mutations in the TARDBP 3′UTR, which block TDP‐43 from binding to its own transcript.^45^, ^56^ Most studies indicate that miRNAs typically attach to a particular sequence located at the 3′UTR of their target mRNAs. This attachment leads to translational repression, as well as mRNA deadenylation and decapping.^57^ So, in addition to directly binding to their own mRNAs, SFs can also indirectly autoregulate their expression by regulating the expression of miRNAs, which can eventually bind to the 3′UTR of SFs’ mRNA. This feedback can be widely observed in SRSF1, SRSF2, SRSF7, SRSF10, hnRNP A1, and RBM5.^58^, ^59^, ^60^, ^61^, ^62^, ^63^ For example, the methyl‐DNA binding protein methyl‐CpG binding domain protein 2 (MBD2) has two predominant isoforms, MBD2a and MBD2c. Only MBD2a contains potential binding motifs for miR‐302.^61^ During the pluripotency phase in human pluripotent stem cells (hPSCs), MBD2c induces the expression of miR‐302, which inhibits the translation of MBD2a. Additionally, MBD2c indirectly increases SRSF2 levels, promoting the splicing to MBD2c and establishing positive feedback.^61^ This finding aligns with computational predictions linking miRNAs and AS.^64^

In addition to mechanisms directly interacting with mRNA sequences, epigenetic changes on 3′UTR, especially methylation, can also collaborate with AS to autoregulate protein levels. S‐adenosyl methionine (SAM) plays a crucial role in transmethylation processes in living organisms.^65^ It can donate the methyl group via the methyltransferase‐like (METTL) family to a large variety of substrates including DNA. It has been found that an excessive amount of SAM can install m^6^A (N6‐methyladenosine) modification on conserved hairpin 1 in 3'UTR of MAT2A mRNA, preventing intron splicing and causing nuclear retention. This leads to decreased expression of MAT2A, resulting in lower SAM production as feedback.^66^ Interestingly, recent studies on *C. elegans* have found that m^6^A modification at the invariant AG of the distal 3′SS can lead to the AS‐NMD of SAM genes.^67^, ^68^, ^69^ Taking into consideration that there have been some clear examples where m^6^A plays a role in splicing decisions in humans, this may indicate that m^6^A‐regulated AS is a conservative mechanism to maintain the SAM expression.^70^, ^71^ ^(p20)^ A recent study revealed that m^6^A modification on the 3′UTR of mRNA TARDBP influences the efficiency of autoregulation, and age‐related demethylation on the autoregulatory region in the 3′UTR of TARDBP DNA can increase transcript expression and potentially lead to the early onset of ALS.^28^, ^72^ In summary, the feedback of AS can be subjected to epigenetic regulation.

## 3 POSITIVE FEEDBACK TRIGGERS STATE CHANGES

**FIGURE 2:**
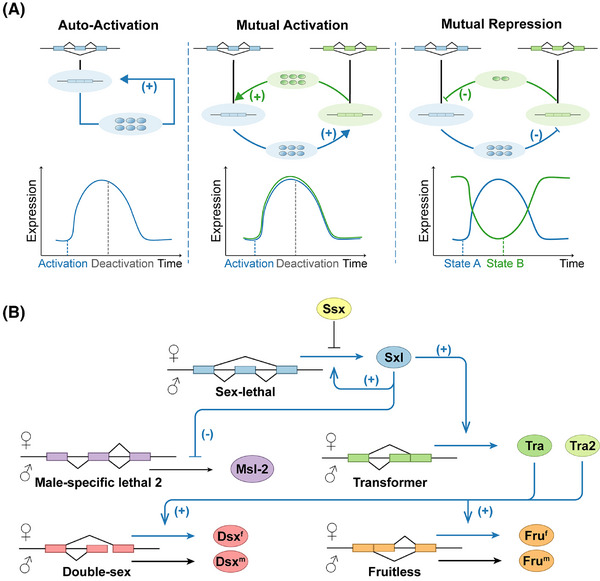
Positive feedback of alternative splicing triggers state changes. (A) Simplified models of positive feedback include one auto‐activating component, two mutual activators, and two mutual repressors. In the line chart below, the horizontal axis represents the triggering, development, and termination of positive feedback; the vertical axis represents the expression level of genes involved in this feedback. (B) Schematic diagram of sex determination in *Drosophila*. Proteins on the upper side of genes are expressed and contribute to the female phenotype, while proteins on the lower side of genes are expressed and contribute to the male phenotype. Triggered by the ratio of X chromosomes to autosomes (X:A), the expression of Sxl can respond in an “all‐or‐none” manner by a positive splicing feedback loop. This influences the splicing pattern of downstream factors, ultimately leading to the differentiation into male or female. The processes influenced by the positive feedback mechanism are highlighted in blue.

Due to SFs playing a greater role in maintaining homeostasis, positive feedback systems are less prevalent compared with negative feedback. However, positive feedback systems still exhibit important characteristics in biological systems such as bistability, hysteresis, and nonlinear activation properties.^53^ Positive feedback occurs when the end product of a process amplifies the initial stimulus, thereby enhancing and reinforcing the continuation of that process. The patterns of positive feedback include one auto‐activating component, two mutually repressing components, and two mutually activating components (see Figure 2A).^73^ Examples of these patterns are listed in Table 1. As the nature of positive feedback is to amplify deviations and trigger state changes, it is common in cell differentiation and cell fate determination.^73^

**TABLE 1:** Examples of positive feedback of alternative splicing^†^

Patterns of positive feedback	Splicing factors	Other proteins involved	Functions	References
Mutual repression	SRSF1	BCL, PIK3C3	Promoting autophagy of gefitinib‐resistant cancer cells	Lv et al. ^74^
	Not identified	NR2F2, OCT4	Activating hESC differentiation	Rosa and Brivanlou ^75^
Mutual activation	RBFOX2	TGF‐β signaling pathway	Promoting neural crest/craniofacial development	Cibi et al. ^76^
	CELF2	MKK7, JNK signaling pathway	Activating T cells	Martinez et al. ^77^
	SFRS2	MBD2	Promoting hPSC self‐renewal and reprogramming.	Lu et al. ^61^
	Not identified	NGF, Shootin1, PI3K signaling pathway	Regulating neuronal Differentiation	Ergin et al. ^78^
	Not identified	CD44v6, Ras	Driving cell cycle progression	Cheng et al. ^79^
	SRSF3	CD44v6, β‐catenin signaling pathway, MDR1, WNT3A	Sustaining drug resistance in colorectal cancer	Ghatak et al. ^80^
	Not identified	CD44v6, YB‐1, MDR1	Sustaining stemness and drug resistance in colorectal cancer	Ghatak et al., ^81^
	ESRP1/2	ZEB1, CDLK2, CD44v6	Promoting epithelial‐mesenchymal transition and breast cancer progression	Li et al. ^82^
	ESRP1	CD44s, ZEB1, hnRNPM	Promoting drug resistance and persistence of EMT in cancer cells	Harvey et al.,^83^ Preca et al.^84^
Auto‐activation	Not identified	G9a	Neural differentiation	Fiszbein et al.^85^
	Not identified	MaMYB16S	Promoting fruit ripening	Jiang et al.^86^
	Not identified	S‐AHNAK	Promoting muscle cell differentiation	de Morrée et al.^87^
	Sxl	‐	Promoting female sex differentiation in *Drosophila*	Cline et al.^88^, Wright et al.^20^
	Cctra	‐	Promoting female sex differentiation in *Ceratitis*	Primo et al.^89^
	Not identified	Tat	Activating HIV‐1 transcription	Likhoshvai et al.^90^

Abbreviations: hESC, human embryonic stem cells; hPSC, human pluripotent stem cells; EMT, epithelial‐mesenchymal transition.

^†^ Examples related to non‐coding RNAs are detailed in Section 5 other than here.

Bistability in a gene regulatory network enables cells to maintain either of two distinct gene expression patterns stably, which can be switched by appropriate inputs.^91^ The sex determination in broad species of insects is well‐known as a notable example of positive feedback and bistability (see Figure 2B).^92^ The sex determination system of *Drosophila* is the most thoroughly studied. The major regulator of this feedback is the sex‐lethal (Sxl) protein. Once received the determination activation signal, which is the ratio of X chromosomes to autosomes (X:A), Sxl can decide whether to express or not.^88^, ^93^ The expression of Sxl can splice its own transcript to skip the male exon, forming a positive feedback loop.^88^ The continually expressed Sxl further leads to the splicing of the transformer (Tra) into a translational transcript, and double‐sex (Dsx^f^) and fruitless (Fru^f^) into the female‐specific transcript, ultimately forming the female morphology.^94^ On the other hand, in chromosomal males, Sxl is not expressed, resulting in the production of the male‐specific isoforms of Dsx^m^ and Fru^m^, along with male‐specific lethal 2 (Msl‐2) that promote the male development.^20^ Unlike *Drosophila*, the chromosomal sex determination system in *Diptera* is male heterogamety, and in *Hymenoptera* it is the haplodiploid system.^95^ Despite this difference, the Sxl‐Tra feedback mechanism appears to be widespread.^96^ It is also noteworthy that Sister‐of‐Sex‐lethal (Ssx) in male *Drosophila* can prevent the accidental activation of the Sxl cascade, by initiating AS‐NMD of Sxl.^97^ M^6^a on the intron of Sxl is essential for the female‐specific splicing of Sxl, and it translationally represses Msl‐2 to prevent unnecessary dosage compensation in females. Absence of Ime4 (m6A methylosome in *Drosophila*) will lead to a sex bias towards maleness and flightlessness.^98^ This indicates the involvement of epigenetic modifications in *Drosophila* sex determination.^98^ The development of a transgene‐based genetic sexing system for the silkworm (*Bombyx mori*) allows for male‐only rearing by utilising the Sxl mechanism.^99^

It is proposed that common driver mutations can stimulate SFs, which in turn regulate various cancer hallmarks.^100^ As a result, SFs can be seen as mediators or amplifiers in these processes. In certain instances, they even function as oncogenes or pseudo‐oncogenes (e.g., SRSF1 and hnRNPA1^101^), fuelling positive feedback that accelerates cancer progression.^100^ Defects in AS are commonly observed in cancer. These defects arise due to mutations in genes encoding cis‐acting elements or SFs, or due to dysregulation in the AS machinery.^7^, ^102^ In studies of AS feedback, abnormal positive feedback enhancement is most commonly observed. Whether or not it is an oncogenic event, positive feedback often promotes the metastasis and growth of cancer. One noble example is CD44, a multifaceted glycoprotein on the cell surface that can undergo splicing to produce various isoforms, directly influencing cancer progression and drug resistance.^103^, ^104^ The pre‐mRNA of CD44 has 10 exons, making it theoretically able to produce over 1,000 different isoforms of the variable region of the CD44 receptor. Its carcinogenic effect is predominantly manifested through its interaction with extracellular matrix and its connection with signal transduction pathways.^104^ An interesting hallmark of cancer is the role of CD44 in the epithelial‐to‐mesenchymal transition (EMT). Research on various cancer cell lines has identified a positive feedback loop between CD44 and ZEB1, which represses ESRP1 gene activity, thereby promoting CD44s splicing and reinforcing EMT.^84^ Additionally, circESRP1 and CTCF can create positive feedback that inhibits the EMT process.^105^ Since the shift in CD44 isoforms is essential for EMT and ESRP1 is a master SF in EMT, it is plausible that CD44s and the depletion of ESRP1 represent a common pathway in the EMT process in cancer.^83^, ^106^ Other examples of positive feedback involving AS are listed in Table 1. Inhibiting some components of the feedback loop has been shown therapeutic potentials in the cancer treatment. For example, D‐JNKI‐1 (XG‐102, currently in phase III clinical trials) can inhibit JNK in breast cancer cell line model, thus breaking the splicing feedback loop of MBNL1‐*MAP2K7*Δexon2‐JNK, leading to a reversal of the tumorigenic dedifferentiation.^107^

## 4 SPLICING CASCADE AMPLIFIES SIGNALS

Splicing cascade refers to a sequence of splicing events that occurs due to a single trigger, leading to message amplification and transfer to effector molecules.^108^ This trigger could be a signalling molecule, environmental stimulus, or any other factor that initiates the splicing process and plays important roles in various processes. AS maintaining hepatocyte homeostasis is one example. Hepatocyte growth factor decreases SRSF3 levels, leading to AS‐NMD of SRSF1, which further affects a variety of splicing events, including that of the tumour suppressor KLF6, resulting in increased cell proliferation.^109^ Myogenesis is another example where the RBM4 antagonises PTBP via AS‐NMD to maximise the production of muscle cell‐specific mRNA isoforms during myogenesis.^110^ Above all, the coordination and regulation of SFs are very delicate, as any disruption can lead to severe diseases, such as cancers. The SR family can participate in multiple splicing cascades, including SRSF3‐MBNL1‐Acin1, RBM4‐SRSF3‐MAP4K4, and RBM4‐Nova1‐SRSF6.^111^ ^¸^ ^112^ These cascades help influence the biological characteristics of colorectal cancer cells, such as metastasis, progression, and apoptosis.^111^, ^112^, ^113^, ^114^

**FIGURE 3:**
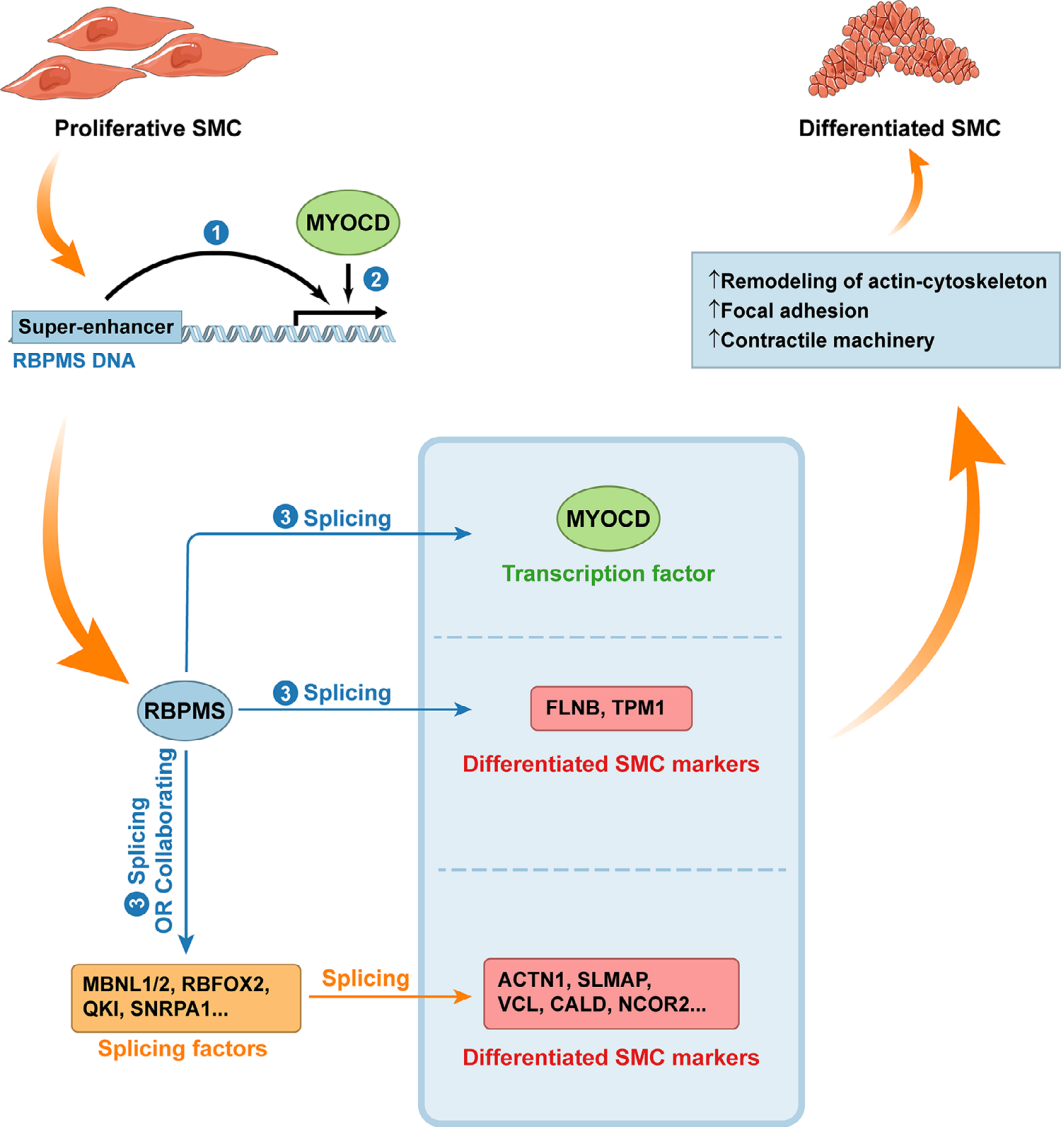
RBPMS acts as a master splicing factor to promote smooth muscle cell differentiation. RBPMS (RNA binding protein, MRNA processing factor) is considered a master splicing factor for several reasons: (1) the transcription of RBPMS is driven by a super‐enhancer. (2) RBPMS receives the regulation of transcription factor MYOCD. (3) RBPMS can directly or indirectly regulate the splicing of a network of proteins, including transcription factors, essential markers in differentiated SMC, and other splicing factors. RBPMS, “RNA Binding Protein, MRNA Processing Factor”; SMC, smooth muscle cells.

A splicing cascade is always featured as a domino effect, where a master splicing regulator initiates subsequent splicing regulations. Master splicing regulators are defined as the SFs that regulate other SFs, usually positioned at the top of splicing cascades, and are essential for cell‐type specification, maintenance, or function.^93^ One criterion for master SFs is that they are regulated independently of the splicing network, such as through transcriptional control or post‐translational modifications.^115^ It is further proposed that the association of their genes with super‐enhancers, which can strongly upregulate important genes, can help identify master splicing regulators.^115^ Therefore, altering the levels of a single SF can significantly reprogram splicing patterns in both normal development and disease.^116^ Master SFs coordinately splice functionally related transcript populations, establishing regulatory networks that enable cells to quickly adjust their transcriptome in response to both intra‐ and extracellular signals.^117^ *RBPMS* (RNA Binding Protein, MRNA processing factor) is a gene coding the master splicing regulator in mammalian smooth muscle cells (SMCs). It regulates the splicing of multiple genes related to cellular functions, including components of the actin cytoskeleton and cell adhesion mechanisms.^115^ The overall effect of RBPMS is to prompt SMCs to progress towards a contractile and mature stage.^115^, ^118^ It is worth noting that RBPMS and KH Domain Containing RNA Binding (QKI), another potent regulator of cellular differentiation, are both closely associated with super‐enhancers and have opposing effects on the splicing of at least two targets, MYOCD and FLNB^115^ (see Figure 3). Besides, RBPMS can interact with several SFs, including MBNL1/2, RBFOX2, and PTBP1, by modifying their isoform ratios or influencing their activity, and these SFs can further regulate the splicing of multiple markers in differentiated SMC, including ACTN1, SLMAP, VCL, CALD, and NCOR2, etc.^115^, ^118^, ^119^ It can also be upregulated transcriptionally by MYOCD or downregulated post‐transcriptionally by phosphorylation, meeting another criterion as a master SF.^115^, ^119^, ^120^ Another prominent master SF is RBFOX2, which controls the expression of many RBPs by regulating silent AS‐NMD events and subsequently modifying their splicing networks.^116^ ESRP1, a master SF in EMT and placental tissues, is shown to be an effective biomarker for immunotherapy of melanoma.^83^, ^121^ MBNL, as a key regulator of the splicing mechanism, can shape large‐scale transcriptome changes and drive cell differentiation.^107^ While a growing number of studies report the identification of new master SFs, clearer criteria are necessary to delineate these findings.

## 5 INTERPLAY OF NON‐CODING RNAS AND SPLICING ESTABLISHES FEEDBACK REGULATIONS

**FIGURE 4:**
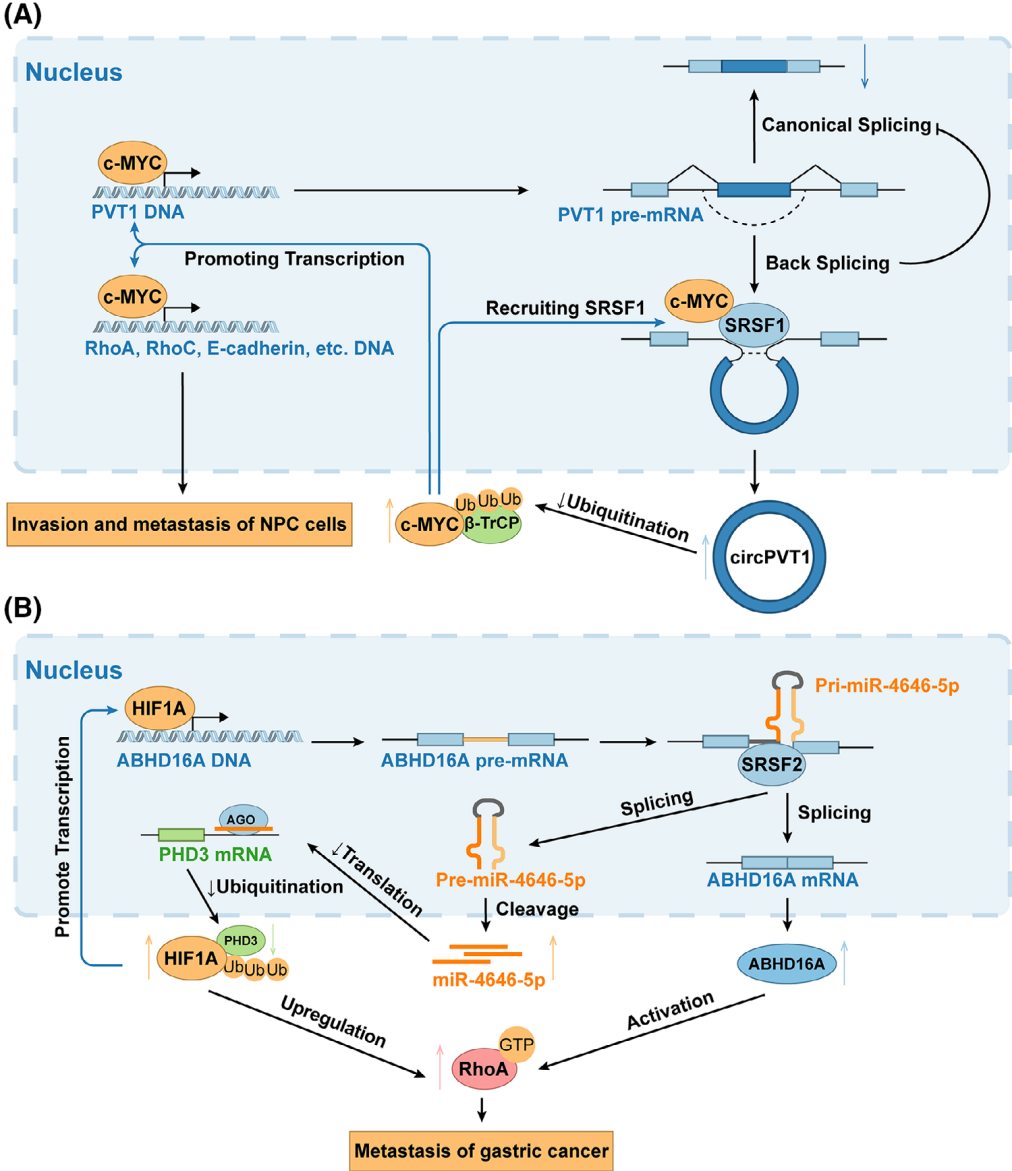
Interplay of non‐coding RNAs and alternative splicing establishes feedback regulations (A) CircPVT1 can prevent c‐MYC ubiquitination and degradation, and c‐MYC further promotes the transcription of PVT1 and recruits SRSF1 to facilitate circPVT1 back‐splicing, establishing positive feedback. This feedback fuels c‐MYC to facilitate the transcription of malignancy‐related proteins including RhoA, RhoC, and E‐cadherin, thereby promoting the invasion and metastasis of NPC cells. The blue strokes indicate the feedback step. NPC, nasopharyngeal carcinoma. (B) SRSF2‐mediated splicing of ABHD16A produces miR‐4646‐5p, which decreases HIF1A ubiquitination by inhibiting PHD3. Elevated HIF1A, acting as a transcription factor, enhances ABHD16A and miR‐4646‐5p expression, creating a positive feedback loop promoting gastric cancer metastasis. HIF1A can also directly bind to the RhoA promoter to regulate RhoA transcription. This synergistic regulation boosts active RhoA levels to promote the metastasis of gastric cancer. The blue stroke indicates the feedback step.

Non‐coding RNAs (ncRNAs), such as circRNAs and microRNAs (miRNAs), play crucial roles in the intricate regulation of AS through feedback loops. These feedback loops involve a complex interplay between ncRNAs, SFs, and mRNA transcripts, ultimately influencing the regulation pathways governing cancer development. CircRNAs are non‐coding RNAs that are generated through a process called back‐splicing. In this process, the 5’ terminus of an upstream exon in a pre‐mRNA is spliced together with the 3’ terminus of a downstream exon, resulting in the formation of a circular molecule.^122^ Given the need for back‐splicing in circRNA formation, it is reasonable to speculate that circRNAs can collaborate with SFs to establish feedback mechanisms. For example, circPVT1 can prevent c‐MYC ubiquitination and degradation, and c‐MYC further enhances the transcription of PVT1 and recruits SRSF1 to facilitate circPVT1 splicing, establishing positive feedback and promoting nasopharyngeal carcinoma (NPC) invasion and metastasis^123^ (see Figure 4A). Other examples of feedback formed by the interplay of circRNAs and splicing are outlined in Table 2. Most of the circRNAs form positive feedback with SFs, promoting the progression of various hallmarks of cancer.^124^ Specifically, the continuously accumulating products of oncogenes almost seem to be byproducts of ongoing positive feedback. The AS‐NMD of FUST can also help with multiple circRNA regulation.^125^

**TABLE 2:** Feedback formed by interplay of circRNAs and Splicing.

CircRNAs	Splicing factors	Other proteins involved	Functions in diseases	References
circARF1	U2AF2	ISL2, miR‐342–3p, VEGFA	Promoting glioma angiogenesis	Jiang et al.^126^
circATP5B	SRSF1	miR‐185‐5p, HOXB5	Promoting glioma stem cell proliferation	Zhao et al.^127^
circCAMSAP1	SRSF10	SERPINH1, c‐MYC	Promoting nasopharyngeal carcinoma tumorigenesis	Yian Wang et al.^128^
circESRP1	CTCF^†^	miR‐3942, c‐MYC	Inhibiting clear cell renal cell carcinoma progression	Gong et al.^105^
circEZH2	FUS	KLF5, miR‐217‐5p, CXCR4	Promoting liver metastasis of breast cancer	Liu et al.^129^
circNCAPG	U2AF65	RREB1, TFG‐β1	Promoting malignant phenotypes of glioma	Li et al.^130^
circNIPBL	ZEB1^†^	miR‐16‐2‐3p, Wnt5a	Promoting metastasis of bladder cancer	Li et al.^122^
circPVT1	SRSF1	β‐TrCP, c‐MYC	Promoting nasopharyngeal carcinoma metastasis	Mo et al.^123^
circROBO1	FUS	KLF5, miR‐217‐5p, BECN1	Promoting liver metastasis of breast cancer	Wang et al.^131^
circSNRK	NOVA1	miR‐33, NF‐κB, Cas3	Regulating cardiac function in post‐myocardial infarction	Wang et al.^132^
circUHRF1	ESRP1	miR‐526b‐5p, c‐MYC, TGF‐β1	Promoting oral squamous cell carcinoma tumorigenesis	Zhao et al.^133^

^†^ Not generally considered as splicing factors, but facilitating the splicing process.

MiRNAs are typically assembled into RNA‐induced silencing complex (RISC), which can guide the degradation of mRNA or inhibit mRNA translation depending on the degree of complementarity between miRNA and target mRNA.^57^ This action primarily takes place in the 3'UTR of target mRNAs, with occasional involvement in the 5' UTR.^7^ It is worth noting that the majority of intragenic miRNAs are located within introns, and they are often transcribed alongside their host genes using the same promoters.^134^ This co‐expression enables another potential for interplay between miRNAs and other proteins. Primary miRNAs (pri‐miRNAs) are traditionally separated from adjacent exons by Drosha; however, some Drosha‐independent miRNAs (known as mirtrons) can achieve this through AS.^57^, ^135^ For example, in Drosha‐low‐expressed gastric cancer cells, SRSF2‐mediated splicing of ABHD16A leads to the abnormal up‐regulation of miR‐4646‐5p. The increased miR‐4646‐5p levels result in a decrease in ubiquitination of HIF1A by inhibiting its target gene PHD3, consequently leading to an elevated HIF1A production. This elevated HIF1A acts as a transcription factor, promoting the expressions of its host gene ABHD16A and miR‐4646‐5p. This constitutes positive feedback to activate RhoA to promote the metastasis of gastric cancer^136^ (see Figure 4B). Other examples of feedback formed by the interplay of miRNAs and splicing are outlined in Table 3. Some SFs can regulate miRNAs by binding to specific sites within the stem‐loop structure of pri‐miRNAs.^58^, ^135^ This binding enhances cleavage by Drosha in a manner independent of splicing, thus promoting the maturation of pri‐miRNAs, such as in the case of miR‐7 and SRSF1.^137^ MiRNAs can act as mediators or fine‐tuners between SFs and target genes in AS feedback.^138^ Evidence has suggested that the same miRNAs can function either as oncogenes or normal expressing genes, depending on the tissue type.^135^ For example, two positive feedback mechanisms, miR‐124/REST/PTB1 and PTB2/BRN2/miR‐9, contribute to the conversion of glial cells into neurons.^139^, ^140^ But these feedback loops also have an impact on the Warburg effect in colorectal cancer.^138^, ^141^ Interestingly, some cases of circRNAs showed that they can take effect by binding to and inactivating miRNAs, a process known as miRNA sponging, thereby upregulating miRNA target gene expression (see some cases in Table 2).^105^, ^126^, ^127^ This phenomenon largely arises from the fact that miRNAs can bind to multiple mRNAs. For example, the circESRP1/miR‐3942/CTCF feedback loop plays a crucial role in inhibiting the progression of renal cell carcinoma.^105^ The genome organiser CTCF specifically promotes the expression of circESRP1, which contains binding sites for miR‐3942. By sponging miR‐3942, circESRP1 prevents miR‐3942 from binding to and inhibiting CTCF mRNA. As a result, the increased levels of CTCF can bind to and repress the transcription of c‐MYC DNA.^105^ This again highlights the intricate network of ncRNAs and splicing. Some other ncRNAs are also found to be involved in AS feedback, including lncRNA,^142^, ^143^ sisRNA^144^ and snRNA.^85^

**TABLE 3:** Feedback formed by interplay of microRNAs and splicing.

MicroRNAs	Splicing factors	Other proteins involved	Functions (in diseases)	References
miR‐4646‐5p	SRSF2	PHD3, HIF1A, ABHD16A	Promoting gastric cancer metastasis	Yang et al.^136^
miR‐30a‐5p, miR‐216b, miR‐181a‐5p	SRSF7	SPP1	Promoting proliferation rate of renal cancer cells	Boguslawska et al.^58^
miR‐7	SRSF1	–	May contribute to SRSF1‐driven tumorigenesis	Wu et al.^137^
miR‐10b‐5p, miR‐203a‐3p	SRSF1	SAM68	Promoting renal cell carcinoma proliferation	Sokół et al.^59^
miR‐183‐5p, miR‐200c‐3p	SRSF2	OCT4, TNFRSF1B, TP53	Inhibiting renal cell carcinoma apoptosis	
miR‐135a‐5p, miR‐149‐5p	hnRNP A1	–	–	
miR‐124	PTBP1	DDX6, E2F1, c‐MYC, PKM1, PKM2	Switching between proliferation and suppression in colon cancer cells	Taniguchi et al.^138^, ^141^
miR‐124	PTBP1	REST, SCP1	Promoting cellular reprogramming to the neuronal lineage	Xue et al.^139^
miR‐9	PTBP2	BRN2	Promoting cellular reprogramming to the neuronal lineage	Xue et al.^145^

^†^ Some cases involving both circRNA and miRNA are summarised in Table 2.

## 6 COORDINATIVE FEEDBACK MODULATES THE CELLULAR STATE BETWEEN TRANSITION AND STABILISATION

According to Waddington's view of cell differentiation, transitions between different cell phenotypes, induced by external stimuli or noise, represent cell fate decisions, with each stable steady state of a gene regulatory network being associated with a particular cell phenotype.^146^ In principle, any cellular state changes or cellular reprogramming would require disrupting the existing homeostatic state and establishing a new sustainable state, which likely involves sequential switches in feedback controls.^145^ Although cellular state changes are primarily driven by positive feedback, this can pose challenges, such as unintentional transitions that can compromise the cell's adaptive behaviour, resulting in cells becoming “excessively stable” rather than “dynamically stable”.^147^ Therefore, a suitable addition of negative feedback can prevent the erroneous activation of positive feedback and simultaneously maintain the dynamic stability of cells.

### 6.1 Splicing orchestrates cell fate decision

**FIGURE 5:**
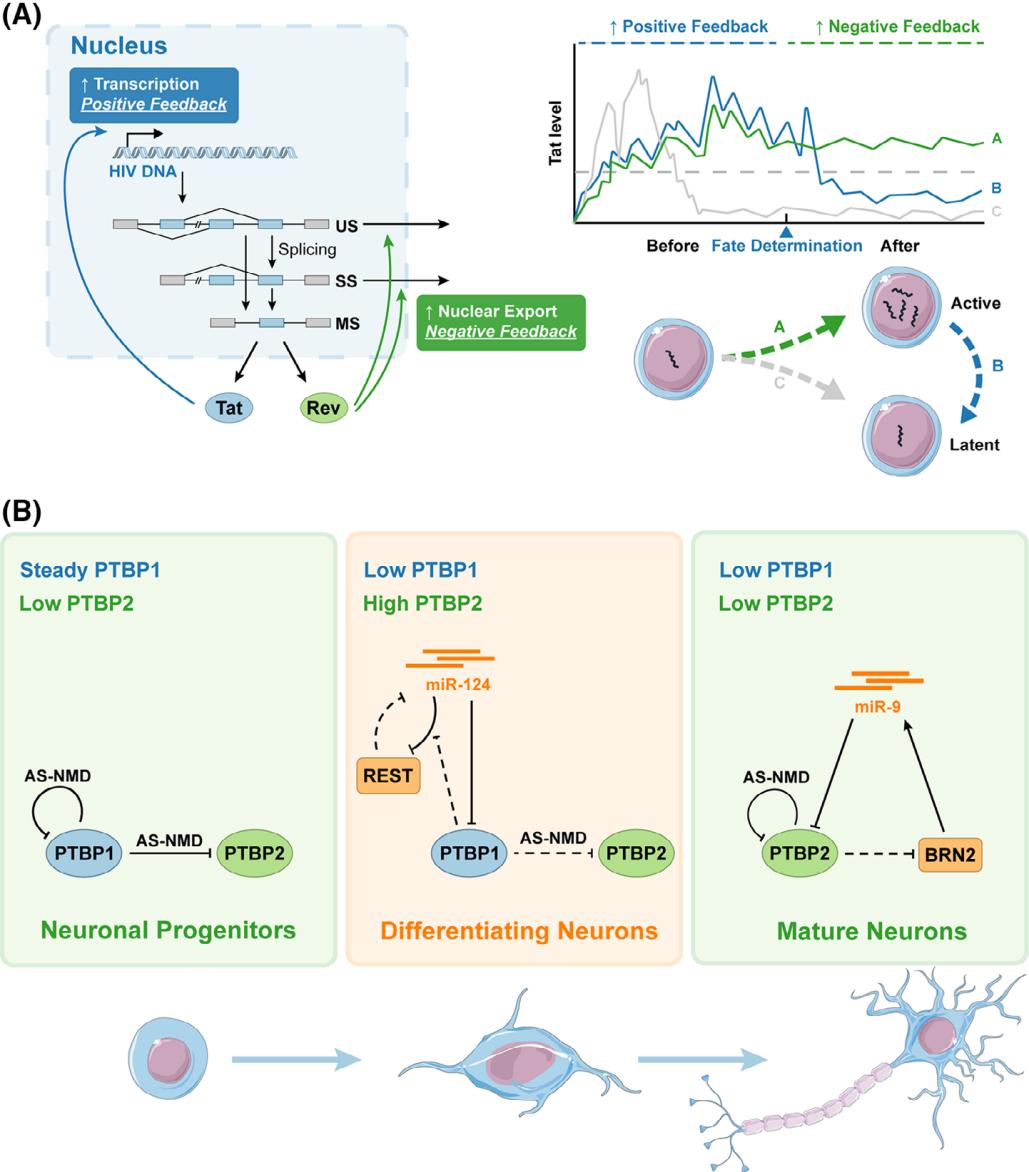
Coordination of feedback modulates and stabilises cellular state. (A) Mechanism of Tat/Rev feedback loops. (Left) The HIV genome is transcribed from the LTR promoter. The primary transcript undergoes post‐transcriptional splicing into various single‐stranded RNA classes: unspliced (US), singly spliced (SS), and multiply spliced (MS). MS RNAs encode early regulatory proteins (e.g., Tat, Rev) and are efficiently exported. Tat protein can enhance transcription (positive feedback loop). Rev protein can bind the element on US and SS RNAs, mediating nuclear export of them. This export constitutes a negative feedback loop via precursor auto‐depletion, reducing the nuclear precursor pool available for splicing into MS RNA. (Right) Tat's expression is essential to determine HIV's fate. Early Tat positive feedback amplifies transcriptional noise, leading to variable Tat levels and probabilistic commitment to either an active (high Tat, lines A/B) or latent (low Tat, line C) state. Following commitment, the Rev negative feedback loop attenuates noise, stabilising the active replication state (line A) and preventing noise‐driven reversion to latency (line B). US, 9‐kb unspliced transcript; SS, 4‐kb singly spliced transcript; MS, 2‐kb multiply spliced transcript. (B) The PTBP family mediates neuron differentiation and maturation. (Left) In neural progenitor cells, PTBP1 is predominantly expressed, playing a critical role in maintaining its own expression and suppressing PTBP2 expression via AS‐NMD. (Middle) During neuronal differentiation, PTBP1 expression is directly downregulated by an increase in miR‐124 levels. This downregulation weakens PTBP1's ability to enhance REST‐mediated inhibition of miR‐124, thereby initiating positive feedback. This feedback mechanism reduces the inhibitory effect of PTBP1 on PTBP2, leading to a marked increase in PTBP2 expression. (Right) In mature neurons, elevated levels of BRN2 and miR‐9 contribute to the inhibition of PTBP2. This inhibition lessens PTBP2's suppressive effect on BRN2, creating positive feedback. Together with AS‐NMD targeting PTBP2, these mechanisms lead to a decrease in PTBP2 levels in mature neurons.

Previous sections have illustrated examples of cell fate decisions driven solely by negative or positive feedback, for example, the negative feedback of polarity and PAR‐5 3’UTR selection in *C. elegans*,^10^ and the positive feedback of sex determination in insects. Yet systems involving a combination of positive and negative feedback can allow for more complex behaviours, particularly in managing noise in gene expression. Noise, the inherent stochastic fluctuation in gene expression, contributes to phenotypic variability and drives cell fate selection.^148^, ^149^ An example highlighting the interplay of feedback, noise, and stabilisation is the transition between latent and active states in the human immunodeficiency virus (HIV). HIV utilises a single promoter to generate a primary transcript that undergoes AS, yielding unspliced (US, ∼9kb), singly spliced (SS, ∼4kb class), and multiply spliced (MS, ∼2kb class) mRNAs (see Figure 5A). Initially, HIV leverages transcriptional noise, amplified by a positive feedback loop involving the Tat protein (encoded by MS transcripts), to facilitate the probabilistic decision between latency and active replication.^150^, ^151^ Tat acts as a potent transactivator binding the TAR element on all nascent transcripts, dramatically increasing overall transcription from the viral LTR promoter. However, for productive replication, a temporal switch in protein expression is essential. In the early phase, only the MS mRNAs, encoding regulatory proteins like Tat and Rev, are efficiently exported from the nucleus and translated. Once sufficient Rev protein accumulates, it binds the Rev response element (RRE) present on US (encoding Gag structural proteins and Pol enzymes) and SS (encoding the Env structural protein and accessory proteins) mRNAs, mediating their export to the cytoplasm via the CRM1 pathway.^150^, ^151^ This Rev‐mediated export of its own precursor transcripts (US and SS are precursors to MS via post‐transcriptional splicing) constitutes a potent negative feedback loop through precursor auto‐depletion.^151^ Crucially, after the fate decision towards active replication is made, this Rev negative feedback strongly attenuates the transcriptional noise amplified by Tat, thereby stabilising the commitment to the active state and ensuring the sequential production of the full suite of viral proteins necessary for virion assembly.^150^, ^151^ Besides the noise arising from random fluctuations in biological processes, regular and periodic oscillations resulting from interactions of feedback can also have a significant impact. Specifically, when a longer, slower positive feedback contains a shorter negative feedback, autonomous oscillations may be generated, leading to biphasic responses.^91^ These responses can either return to the initial state (more likely when the signal is short‐lasting) or transition to another steady state (more likely when the signal is persistent).^91^ The system of Tat and Rev appears to align with this model, although further validation is needed.

Coordinated feedback is also crucial for maintaining specific functional states required for a cell's identity and role, which could be complicated when involving multiple isoforms of the same protein or protein family. The Quaking (Qk) gene exemplifies this, utilising AS to produce functionally distinct isoforms essential for processes like oligodendrocyte differentiation and myelination.^152^ The nuclear Qk5 isoform is essential for splicing regulation and promotes the accumulation of all Qk transcripts.^153^ Qk5 also directs AS of the Qk pre‐mRNA to favour the cytoplasmic Qk6/Qk7 isoforms (translation/stability).^152^ This splicing feedback, combined with Qk5's negative autoregulation (via 3'UTR) and cross‐regulation by Qk6 (promoting its own translation, repressing Qk5's), forms an intricate network balancing nuclear and cytoplasmic activities.^153^ This system achieves a homeostatic, cell‐type‐specific ratio of Qk isoforms, ensuring the cell maintains the correct balance of post‐transcriptional regulation needed for its specialised functions.

Autophagy, the natural degradation of unnecessary or dysfunctional cellular contents, is another important event in cell fate. It is critical in minimising oxidative stress and preventing protein and DNA damage. Therefore, autophagy is often considered as a tumour‐suppressive mechanism.^74^ Different isoforms often exhibit antagonistic effects on autophagy Different isoforms often exhibit antagonistic effects on autophagy, that is pro‐autophagy isoforms (BECN1, ATG14L, ATG12L, LAMP2B, and LAMP2C) contrasting anti‐autophagy isoforms (BECN1S, ATG14S, ATG12S, LAMP2A).^154^ This aligns with our assumption that splicing isoforms typically counterbalance each other to maintain homeostasis, or to facilitate cellular fate switching. In lung cancer cells, a feedback mechanism involving SRSF1 drives the splicing switch of Bcl‐x from the short isoform to the long isoform under normal conditions.^74^ However, in starvation‐induced autophagy, the degradation of SRSF1 leads to the splicing of Bcl‐x transcripts into shorter isoforms. As a result, Bcl‐xS is unable to inhibit autophagy, leading to further degradation of SRSF1 and eventually cell death.^74^

### 6.2 Polypyrimidine tract‐binding protein mediates neuronal differentiation

SFs within the same family often influence each other, akin to the previously mentioned isoform homeostasis. For instance, when one protein is depleted, it can trigger the expression of another family member, which is a prevalent occurrence in the SR family.^155^ PTBP, which has three paralogs in vertebrate genomes, can be found in different tissues and cell types. PTBP1 is expressed in neuronal progenitor cells and a wide range of cells outside the nervous system.^156^ On the other hand, PTBP2 is specifically found in neurons and is commonly referred to as nPTBP or brain‐specific brPTBP.^157^ Numerous studies have demonstrated the vital involvement of PTBP1 and PTBP2 in neurogenesis.^140^, ^158^ Neuronal differentiation can be classified into three phases: neuronal progenitors, differentiating neurons, and mature neurons (see Figure 5B). During these phases, the expression of the two PTBP proteins changes, with PTBP1 peaking in the first phase and PTBP2 peaking in the second phase, eventually both proteins being decreased in the third phase.^159^ This is important because PTBP1 and PTBP2 have distinct effects on the AS of sensitivity‐specific transcript exons; that is, some exons are more repressed by PTBP1 than PTBP2, and vice versa. This provides insight into how proteins respond to different developmental stages.^139^, ^156^, ^160^ The mechanism underneath involves that both PTBP1 and PTBP2 can regulate their own expressions through AS‐NMD.^157^, ^161^ Additionally, PTBP1 can repress PTBP2 via AS‐NMD. Given that PTBP2 is responsible for inducing neuronal‐specific splicing programs, this may account for the decreased PTBP2 levels observed in non‐neuronal cells dominated by PTBP1.^162^, ^163^ Similar processes occur during the reprogramming from glial cells to neurons, especially in humans.^164^ Xue et al. have discovered that repression of PTBP1 can relieve PTBP‐mediated blockage of miRNA action on multiple components of the REST complex, thereby derepressing a large array of neuronal genes.^139^ However, it is not enough to trigger neuronal maturation unless PTBP2 is suppressed to release the inhibition on the transcriptional activator BRN2 and miR‐9.^145^ Overall, reprogramming or converting resident glial cells into neurons in vivo offers a promising strategy for neural regeneration following central nervous system injury.^140^

## 7 CONCLUSION AND PERSPECTIVES

AS is an intricate dance of molecular interplay, where events not only occur in isolation but significantly influence one another. In this review, we have highlighted representative mechanisms governing the autoregulation of SFs, exploring both the negative and positive feedback loops. The complexities extend further to the mutual regulation among SFs and the pivotal role of non‐coding RNAs, serving as either mediators or fine‐tuners of these intricate processes. Lastly, we focus on two cases involving fate determination, HIV and the PTBP family, to illustrate how feedback loops of AS can collaborate consecutively to facilitate shifts in cellular states. These profound interconnections highlight the biological significance and potential implications, particularly in diseases related to splicing abnormalities. As we synthesise these insights, we further discuss the future directions and therapeutic possibilities that could transform AS research and treatment, emphasising emerging methods and challenges for advancing our understanding of AS dynamics.

### 7.1 Expanding research directions in splicing mechanisms and feedback systems

Our growing understanding of AS mechanisms prompts several promising research directions to distill the vast biological information into discernable mechanisms that can be generalised and applied.^93^ The depth and breadth of information pose both opportunities and challenges, with one of the primary tasks being to condense this complex knowledge into broad biological concepts. First, beyond canonical negative and positive feedback loops, additional feedback mechanisms might govern AS regulation. For example, there may be “dual” feedback loops that combine features of both types or “context‐specific” feedback loops that vary by cell type or environmental condition. Dissecting these mechanisms across tissues or under varying physiological states could reveal unique splicing pathways that either promote cellular resilience or lead to dysfunction.^165^ Since alternative transcripts are often specific to tissues or cell types, single‐cell transcriptomics and spatial transcriptomics are expected to offer significant insights into the roles and dynamics of AS.^20^ Single‐cell transcriptomics can capture transient or rare AS events that bulk RNA‐seq would miss, and spatial transcriptomics enables researchers to examine AS in its native spatial context, making it possible to study how microenvironments influence splicing. These technologies could uncover rare cell populations or local cellular environments where novel AS mechanisms are active, leading to more nuanced models of splicing regulation. However, accurately quantifying AS in single cells remains a technical and computational challenge and therefore expands new research directions.^20^ These potential researches are expected to play a transformative role in AS research, especially in fields such as oncology and neurology, where tissue‐specific splicing patterns are often linked to disease.

### 7.2 Enhancing therapeutic strategies: antisense oligonucleotides and beyond

From a therapeutic standpoint, insights into AS feedback systems provide exciting opportunities for intervention in splicing‐related diseases. Antisense oligonucleotides (ASOs) and small molecules already show promise for modulating splicing in disorders. ASOs have emerged as a promising therapeutic modality to modulate AS in genetic disorders. ASOs are short, synthetic strands of nucleotides that can specifically target pre‐mRNA to either promote or inhibit splicing of particular exons. This approach has demonstrated success in diseases such as spinal muscular atrophy (SMA), where the ASO therapy has been approved to restore functional SMN protein levels by modifying SMN2 splicing.^166^ By targeting specific SF autoregulatory mechanisms, ASOs could be tailored to adjust splicing in a more controlled manner, offering potential for selective therapies in complex diseases, including cancers where splicing dysregulation is prevalent.^167^ Additionally, with increased understanding of how SFs interact and influence one another in cascading networks, future therapeutic approaches might employ “multi‐target” ASOs designed to modulate several SFs simultaneously, producing a more robust regulatory effect in diseased cells.^168^ Small molecules that modulate AS offer an additional therapeutic approach, providing an alternative to ASOs with the advantage of potentially crossing the blood–brain barrier and exhibiting systemic effects. For instance, compounds like risdiplam have shown efficacy in treating SMA by modifying splicing without the need for direct genetic manipulation.^169^ This pharmacological modulation could be applied in other splicing‐related diseases, particularly cancers where certain SFs are overexpressed or mutated.^170^ The development of small molecules targeting specific SFs or splicing pathways is an area of intense research and holds promise for expanding treatment options.

### 7.3 Challenges and opportunities in developing alternative splicing‐targeted therapies and towards precision medicine

Several challenges must be addressed for AS‐targeted therapies to reach their full potential. For instance, our understanding of which SFs qualify as “master regulators” remains limited.^171^ Identifying these master regulators could transform therapeutic strategies, enabling us to prioritise targets with the most significant regulatory impact. Furthermore, questions remain about the role of post‐translational modifications (PTMs) in AS feedback mechanisms. PTMs—such as phosphorylation or acetylation of SFs—may introduce additional layers of regulation that allow cells to fine‐tune splicing responses even further.^172^ Exploring the intersection of AS with PTMs could reveal new drug targets or combination therapies that complement existing ASOs or small‐molecule splicing modulators. Ultimately, the knowledge gained from studying AS feedback systems has the potential to drive a paradigm shift in how we diagnose and treat genetic diseases. With improved methods for AS quantification and real‐time feedback analysis, personalised treatments could be designed to address the specific splicing aberrations present in each patient's cells. For example, by profiling a patient's unique splicing landscape, clinicians might better predict disease progression, select optimal treatment targets, or even preemptively identify high‐risk patients. In oncology, where altered AS is frequently linked to tumour progression, therapies that adjust the splicing dynamics in tumour cells could provide an effective, tailored approach with fewer side effects than conventional treatments.^173^

The promise of AS research, particularly as it relates to SF autoregulation and inter‐regulation, holds significant potential for biomedical advancements. As we deepen our understanding of these complex feedback loops and develop the tools to manipulate them, new therapeutic opportunities and scientific breakthroughs will emerge. A targeted focus on SF interactions, non‐coding RNA involvement, and feedback dynamics could yield actionable insights and, ultimately, bring us closer to realising the full therapeutic promise of AS regulation in precision medicine.

## AUTHOR CONTRIBUTIONS

**Zhonghao Guo** wrote the manuscript and created the figures. **Xurui Zhang** collected relevant literature. **Yachen Li** created the figures. **Yule Chen** and **Yungang Xu** reviewed and revised the manuscript.

## CONFLICT OF INTEREST STATEMENT

The authors declare no conflicts of interest.

## ETHICS APPROVAL AND CONSENT TO PARTICIPATE

Not applicable.
